# Supplementary figures and images for: Aberrant expression of collagen type X in solid tumor stroma is associated with EMT, immunosuppressive and pro-metastatic pathways, bone marrow stromal cell signatures, and poor survival prognosis
Source: BMC Cancer. 2025 Feb 12;25:247. doi: 10.1186/s12885-025-13641-y (PMC11823173; doi:10.1186/s12885-025-13641-y)

A

BRCA

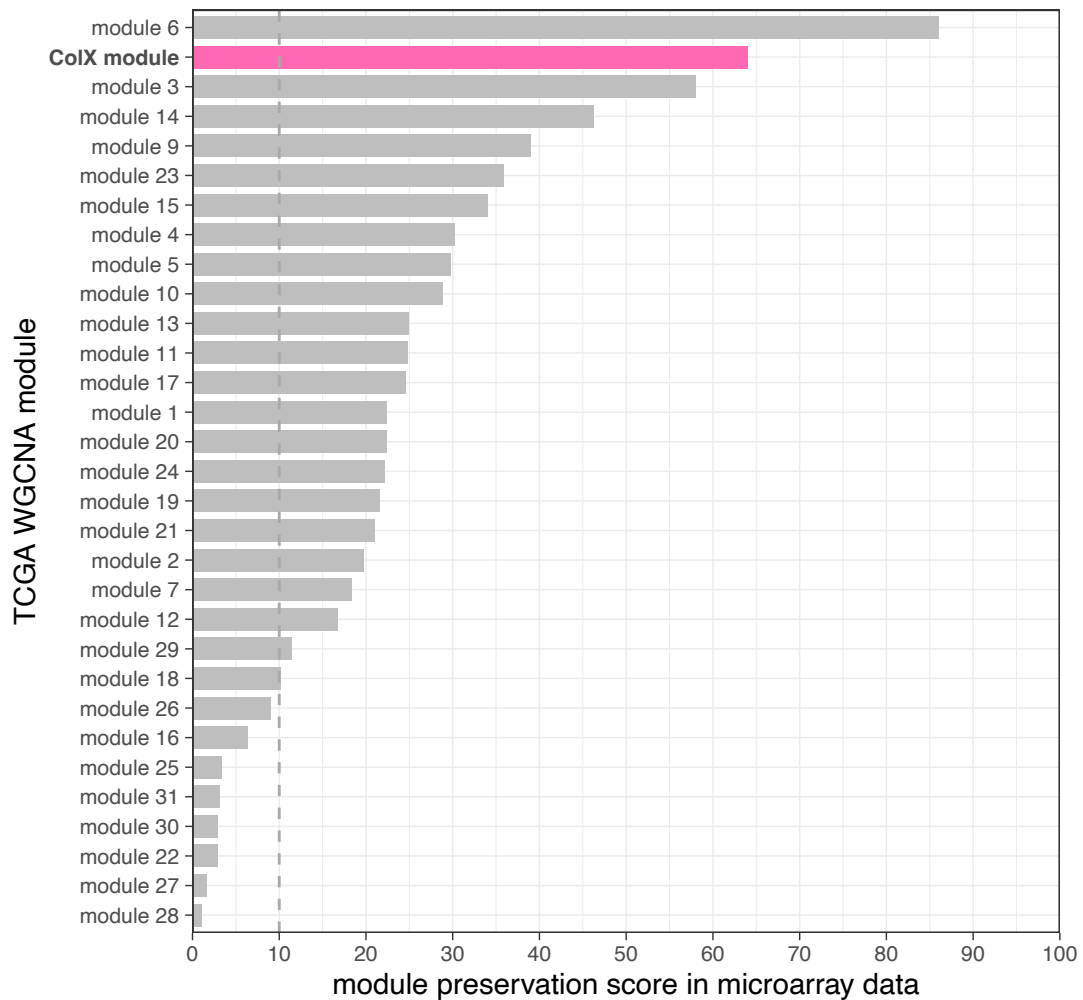

B

PAAD

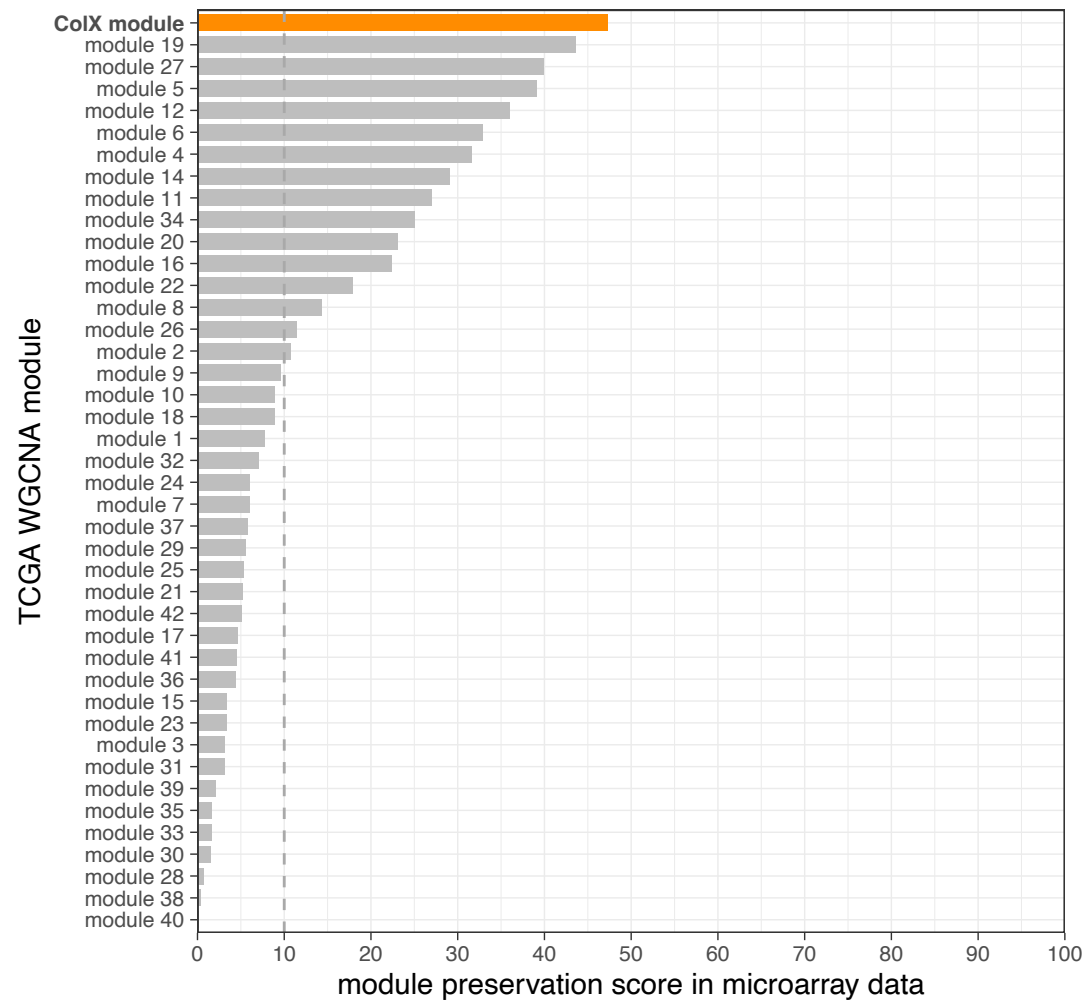

Supplement: Supplementary file 1 — Supplementary Material 1. Figure S1: TCGA ColX modules are preserved in cancer microarray datasets. (A and B) Module preservation scores (Zsummary) for all TCGA RNA-Seq-derived (A) breast and (B) pancreatic cancer WGCNA modules in comparably-sized microarray tumor datasets. ColX modules are indicated by (A) pink or (B) orange bars. Dotted lines indicate “high preservation” threshold of Zsummary = 10 as defined by the authors of WGCNA. [file 12885_2025_13641_MOESM1_ESM.pdf]

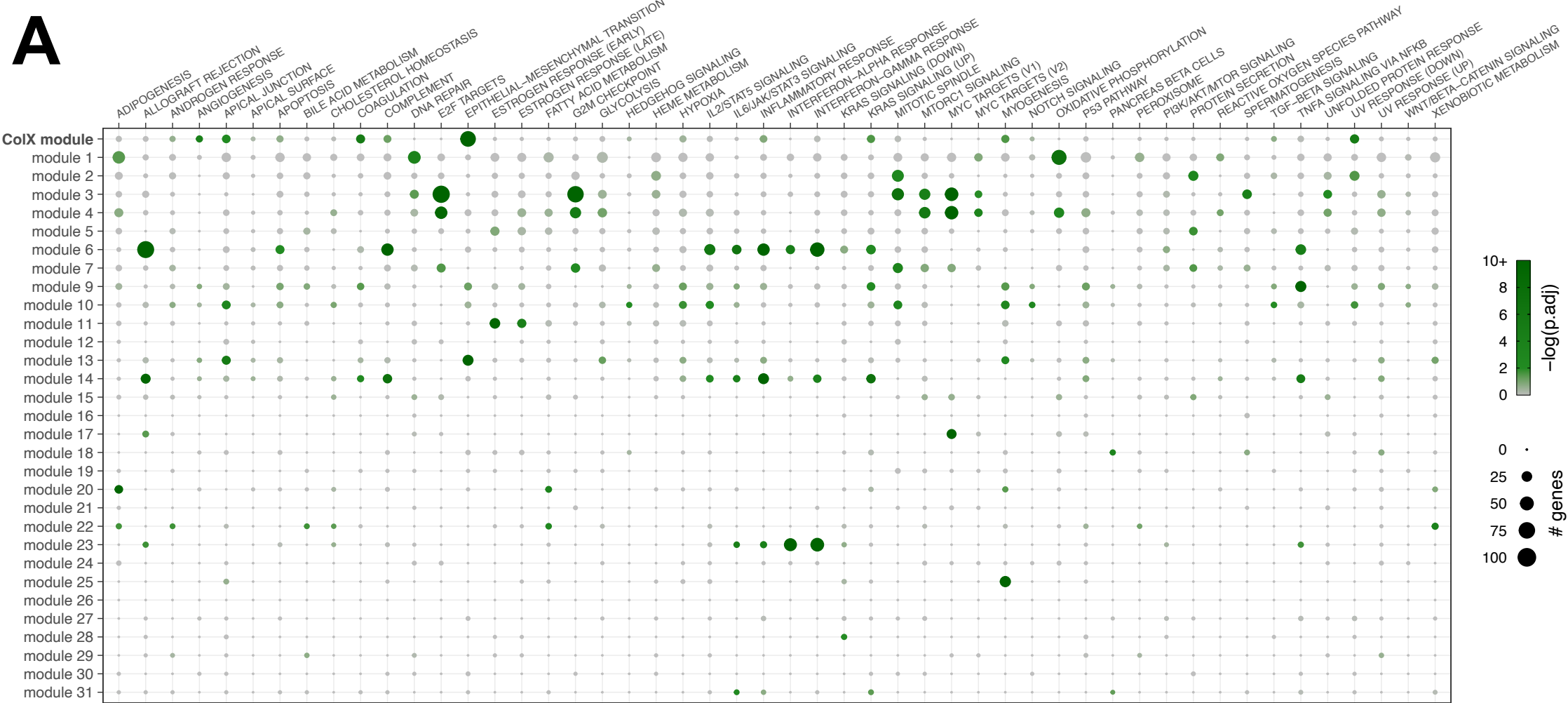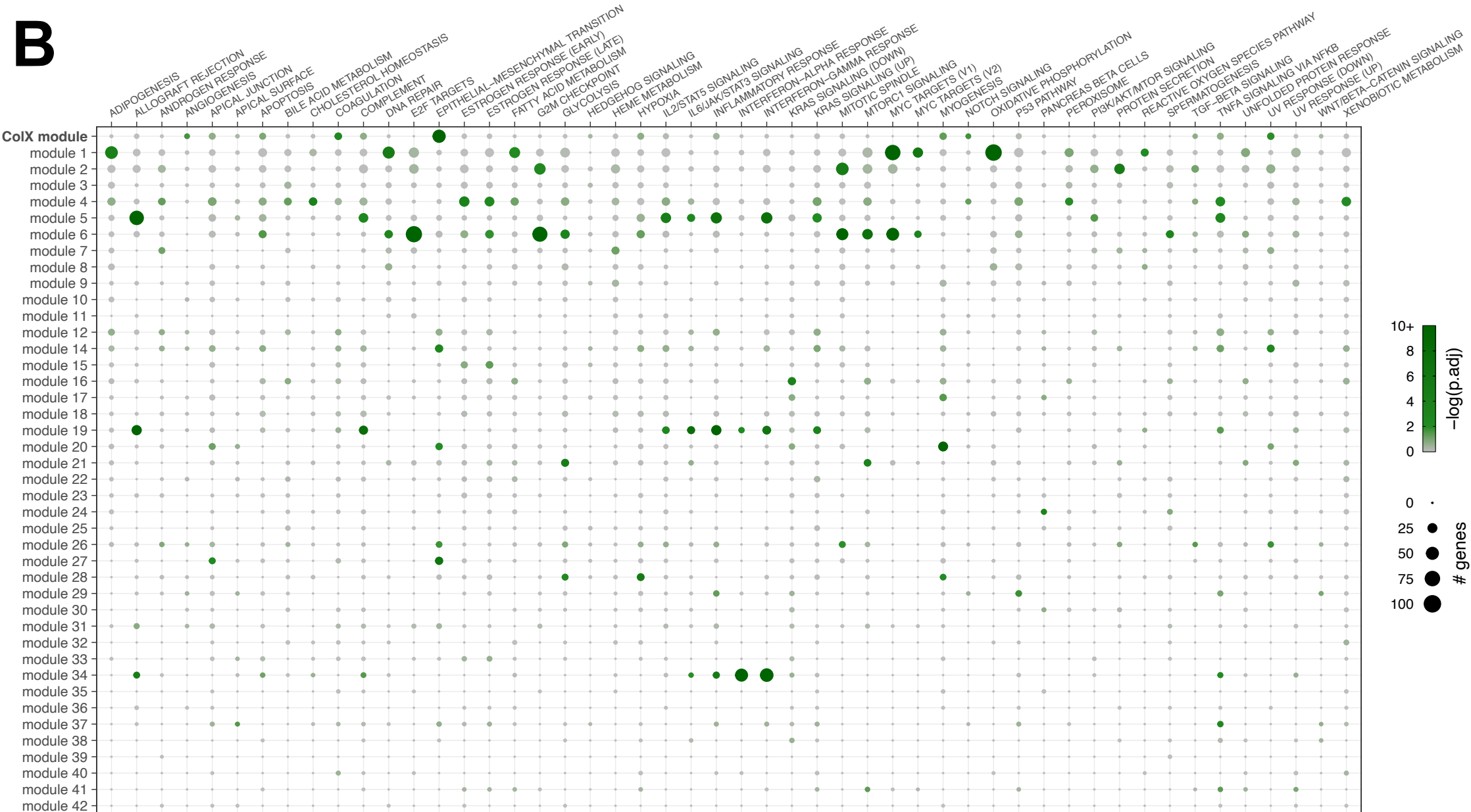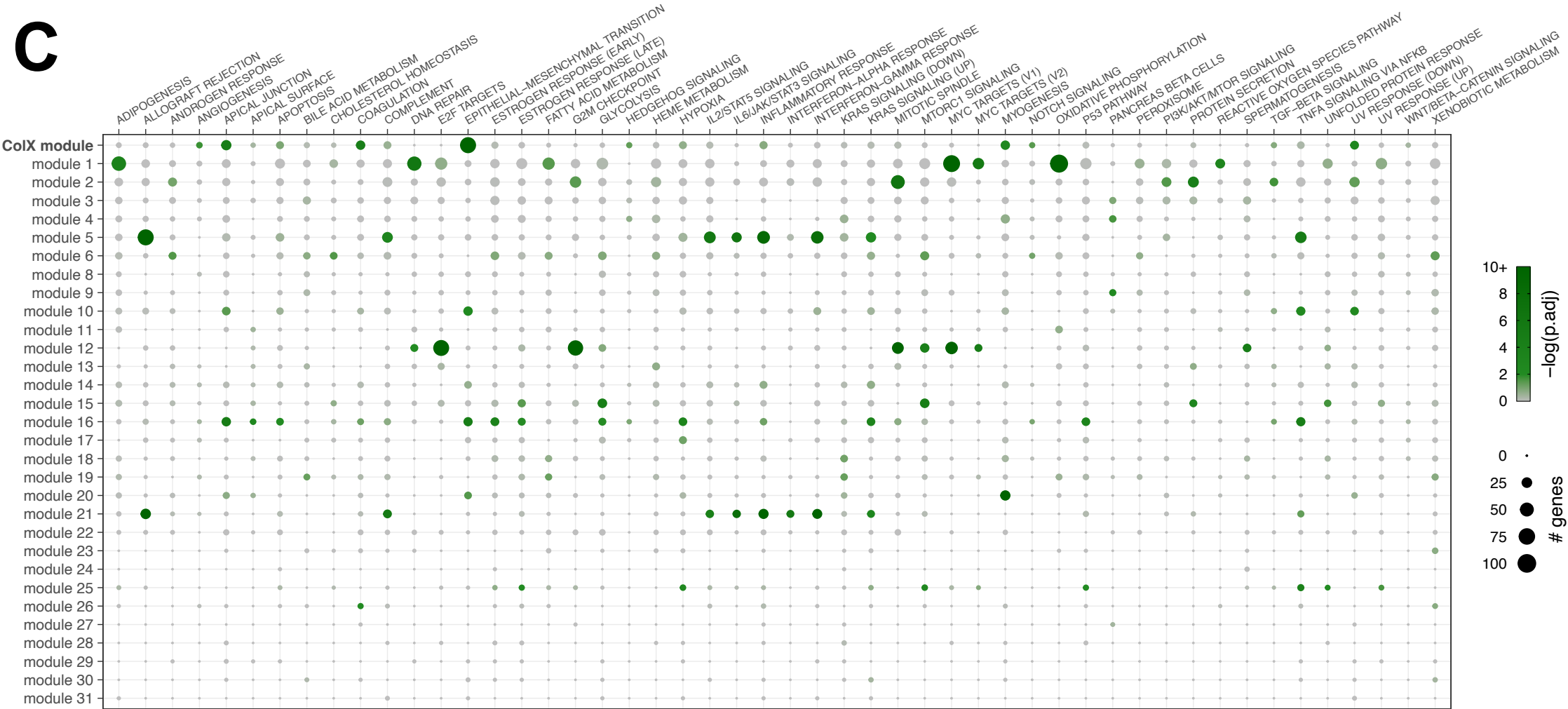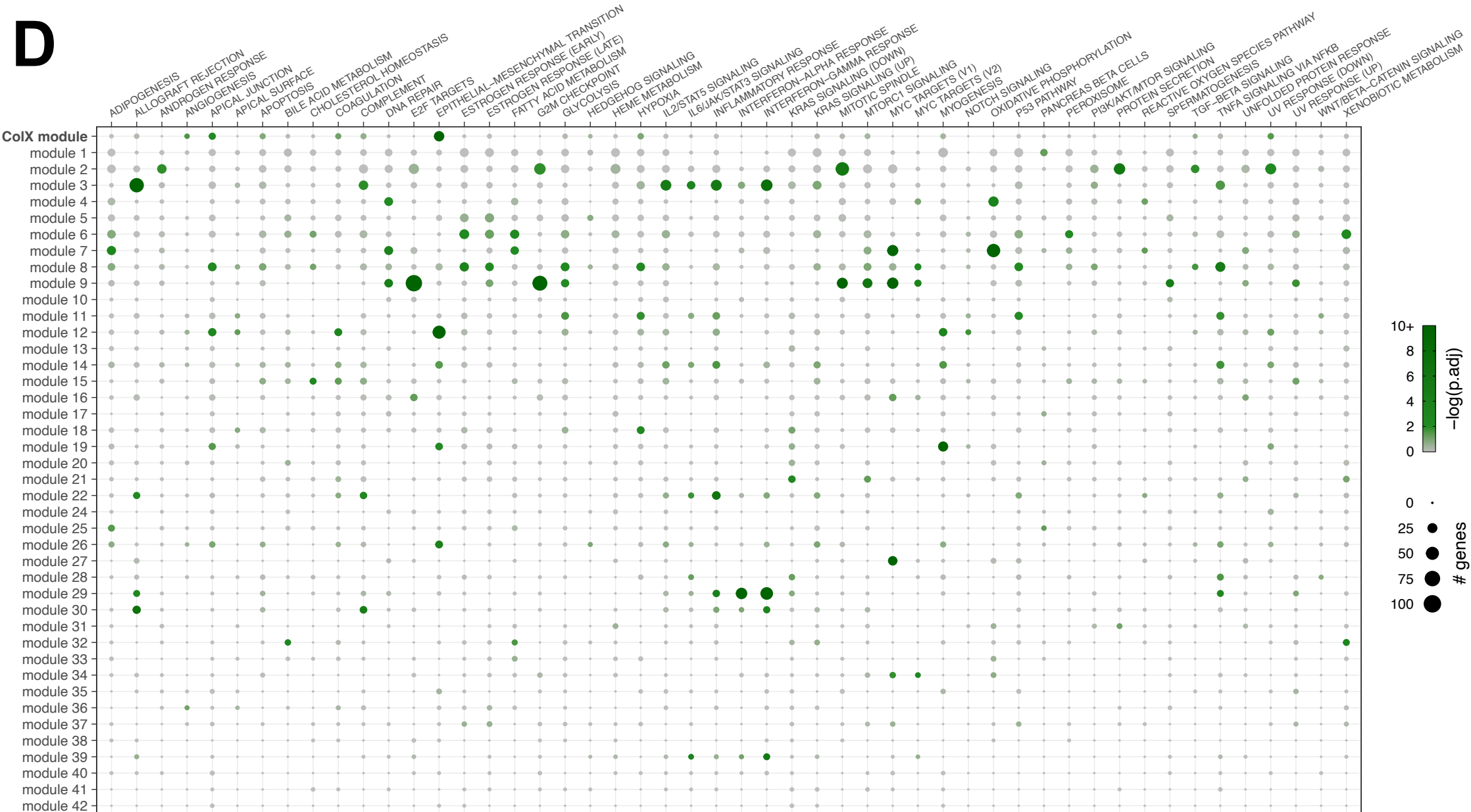

**E**

## EMT Hallmark Pathway Genes in ColX Modules

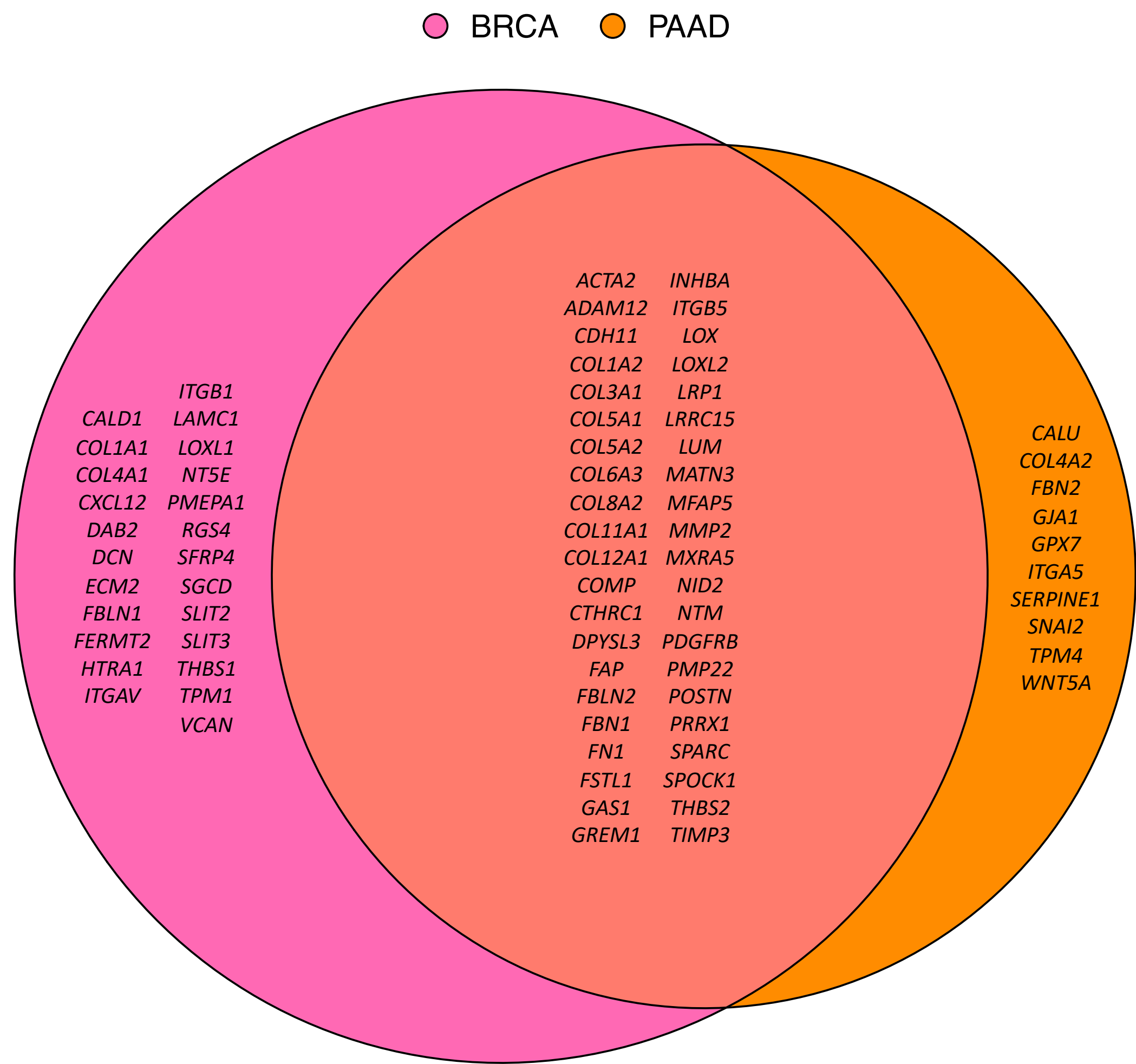

**F**

## EMT Hallmark Pathway Genes in ColX Modules

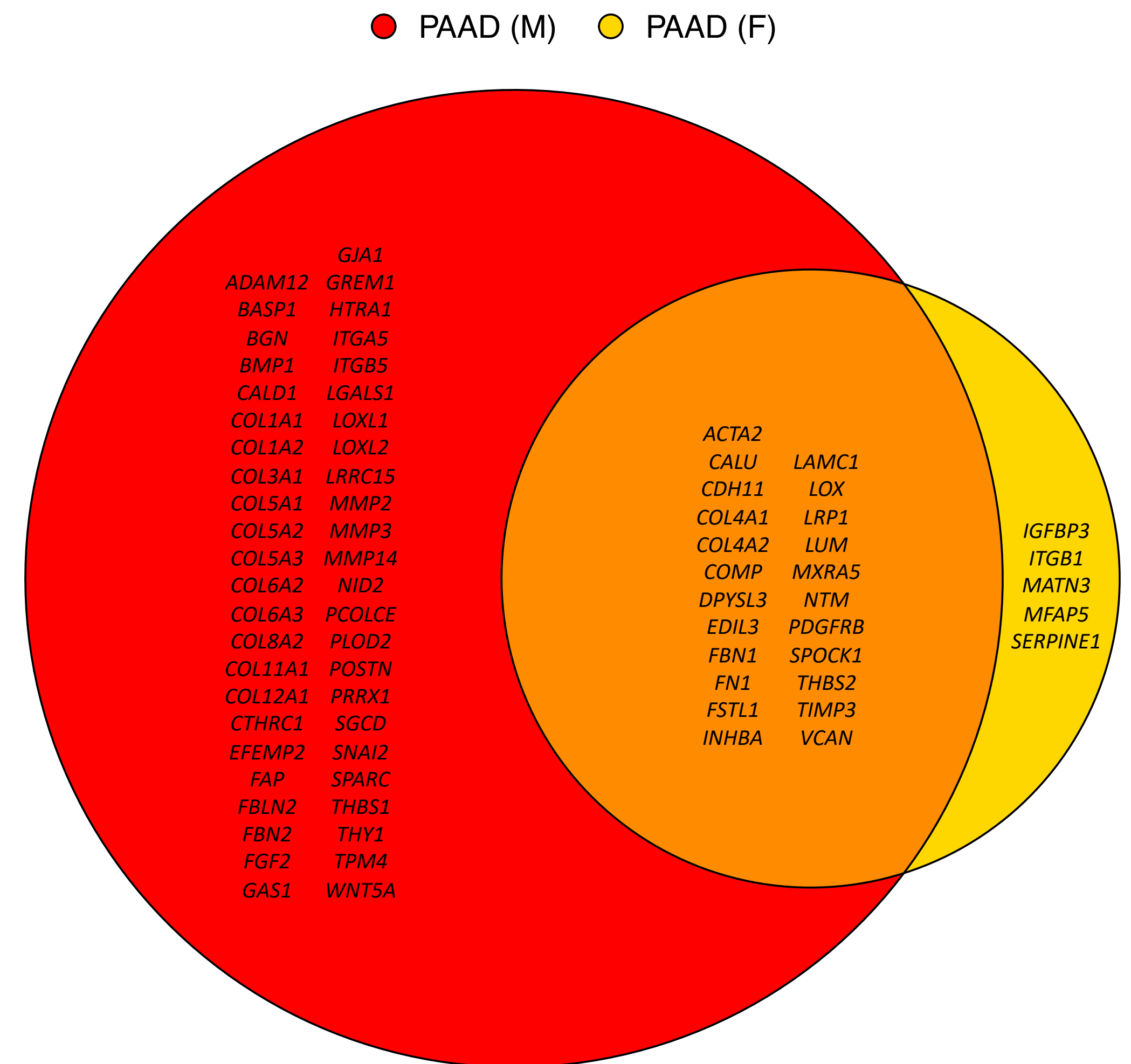

Supplement: Supplementary file 2 — Supplementary Material 2. Figure S2: TCGA WGCNA modules are enriched for numerous hallmark pathways. (A–D) Bubble plots of MSigDB hallmark pathway gene sets [54] enrichment in WGCNA modules from (A) breast cancer, (B) pancreatic cancer, (C) male pancreatic cancer, and (D) female pancreatic cancer cohorts. ColX modules for each dataset are indicated by bolded labels. See Table S4 for significance values and Figure 2D for ColX module-specific enrichment results. (E and F) Overlap of EMT hallmark pathway genes within ColX WGCNA modules from TCGA (E) breast and pancreatic cancer and (F) gender-segregated pancreatic cancer datasets. Genes comprising each sector are listed alphabetically. [file 12885_2025_13641_MOESM2_ESM.pdf]

**A****BRCA**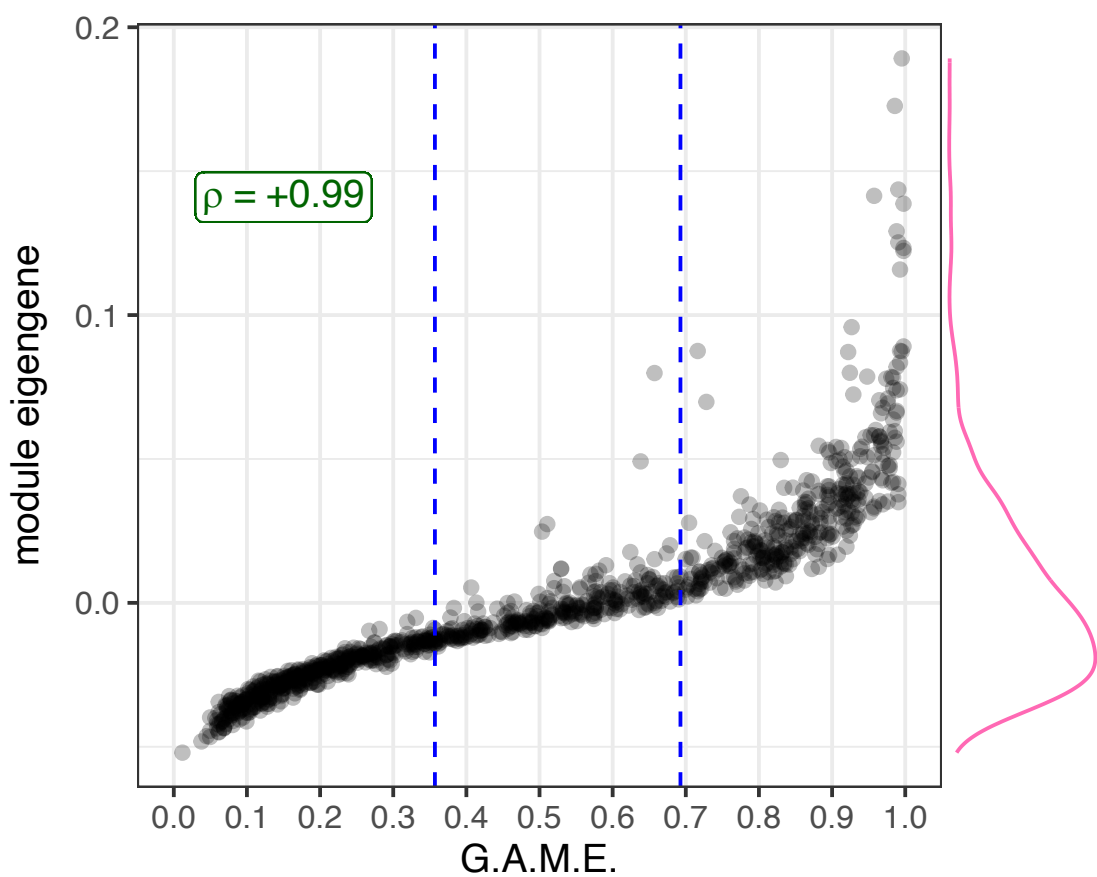**B****PAAD**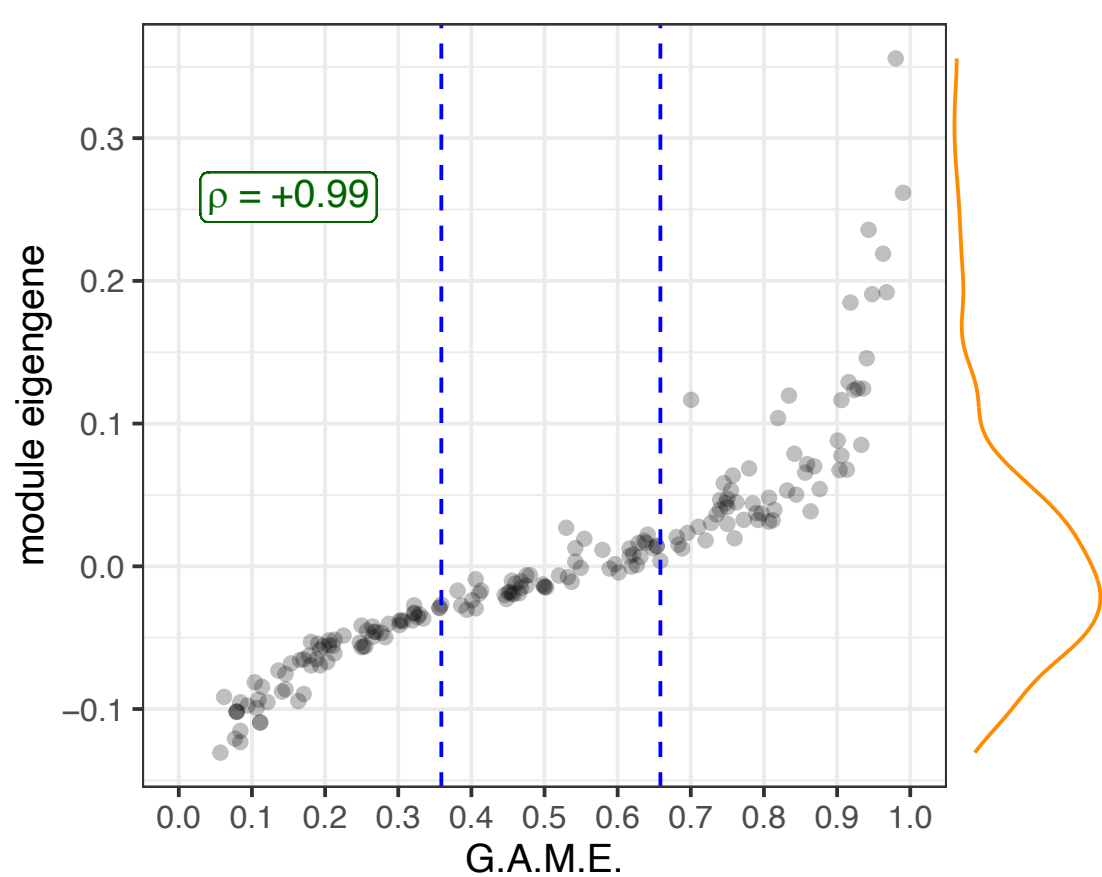**C****PAAD (M)**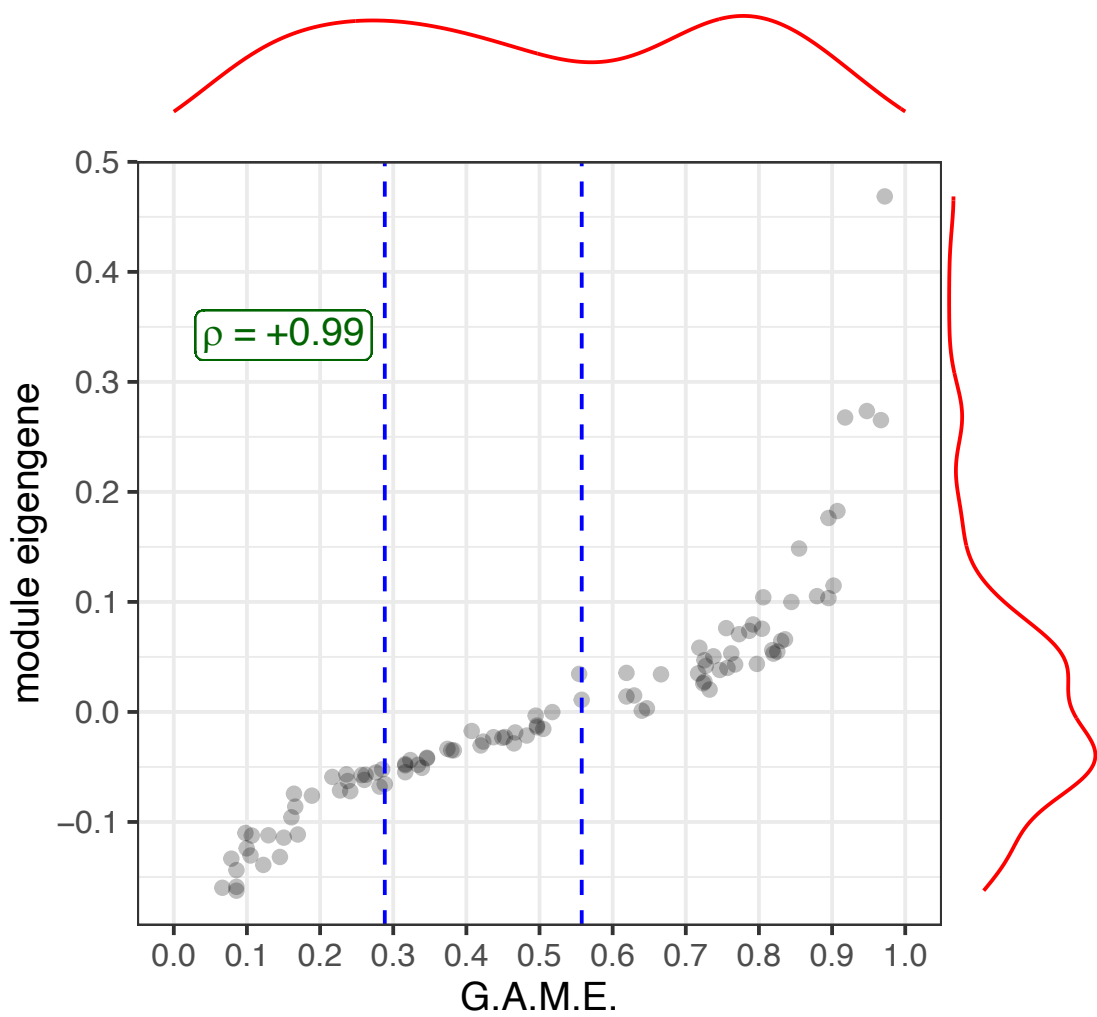**D****PAAD (F)**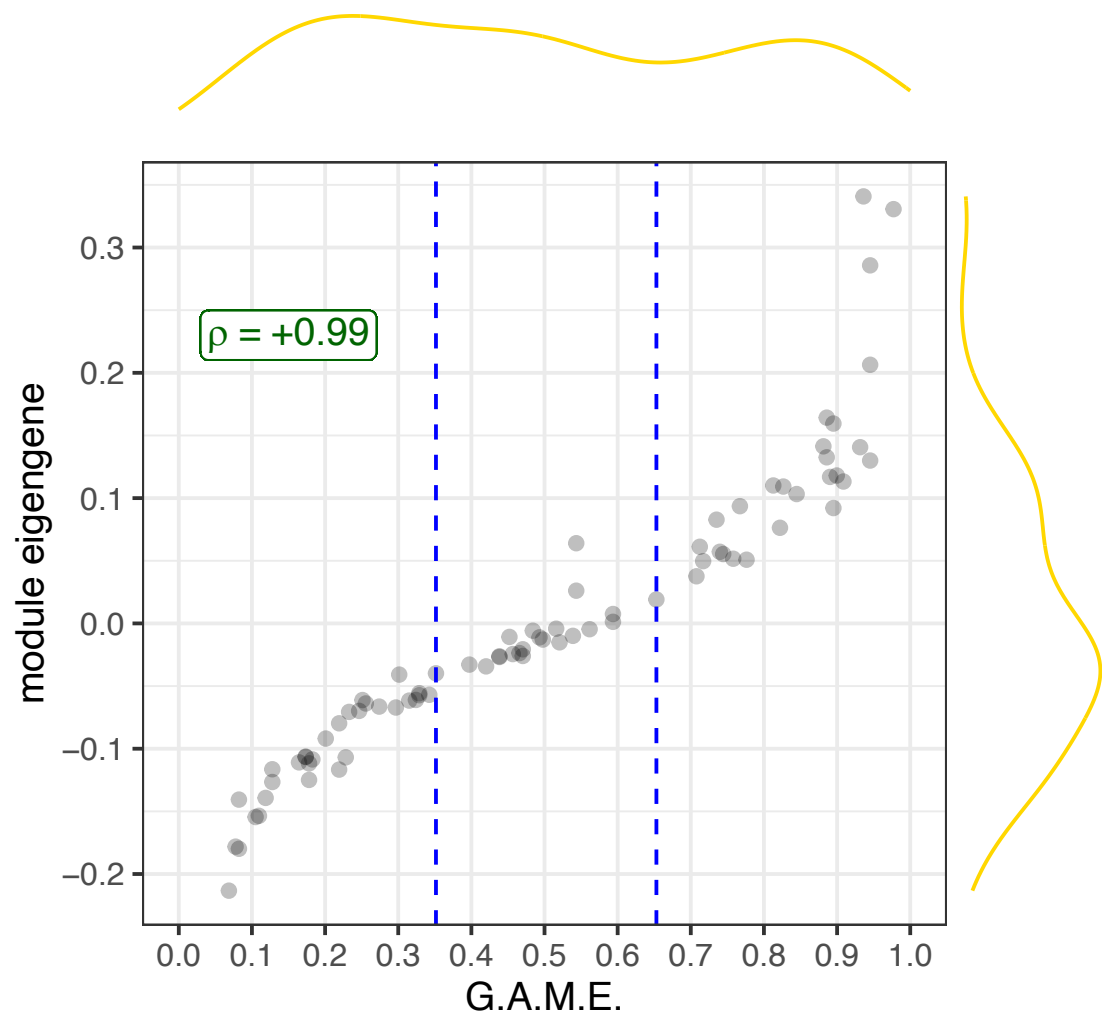

Supplement: Supplementary file 3 — Supplementary Material 3. Figure S3: The G.A.M.E. metric effectively proxies ColX module expression and improves sample stratification. (A–D) Relationship between ColX module eigengene (ME) expression and proportion of “Genes Above Median Expression” (G.A.M.E.) for (A) breast cancer, (B) pancreatic cancer, (C) male pancreatic cancer, and (D) female pancreatic cancer cohorts. Colored curves represent densities of ME (right) and G.A.M.E. (top) variables for each panel. Blue dotted lines indicate Jenks natural breakpoints defining 3 clusters (“low”,“medium”, and “high”). Spearman correlations (ρ) are shown for each panel. [file 12885_2025_13641_MOESM3_ESM.pdf]

**A****BRCA**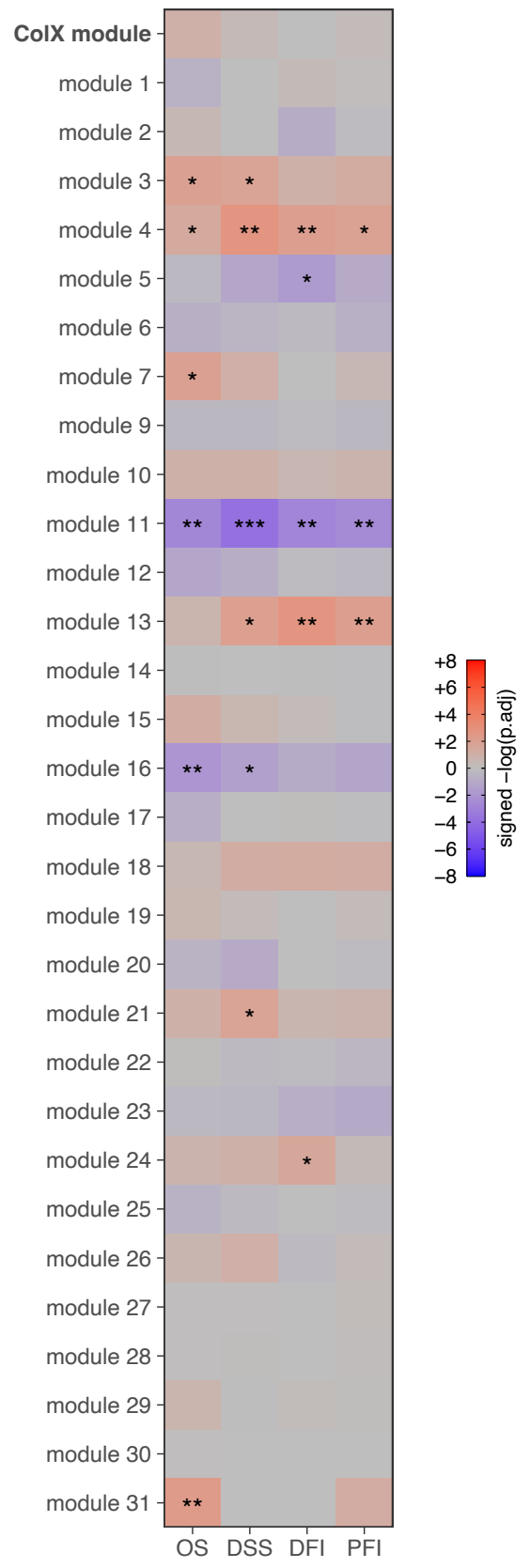**B****PAAD**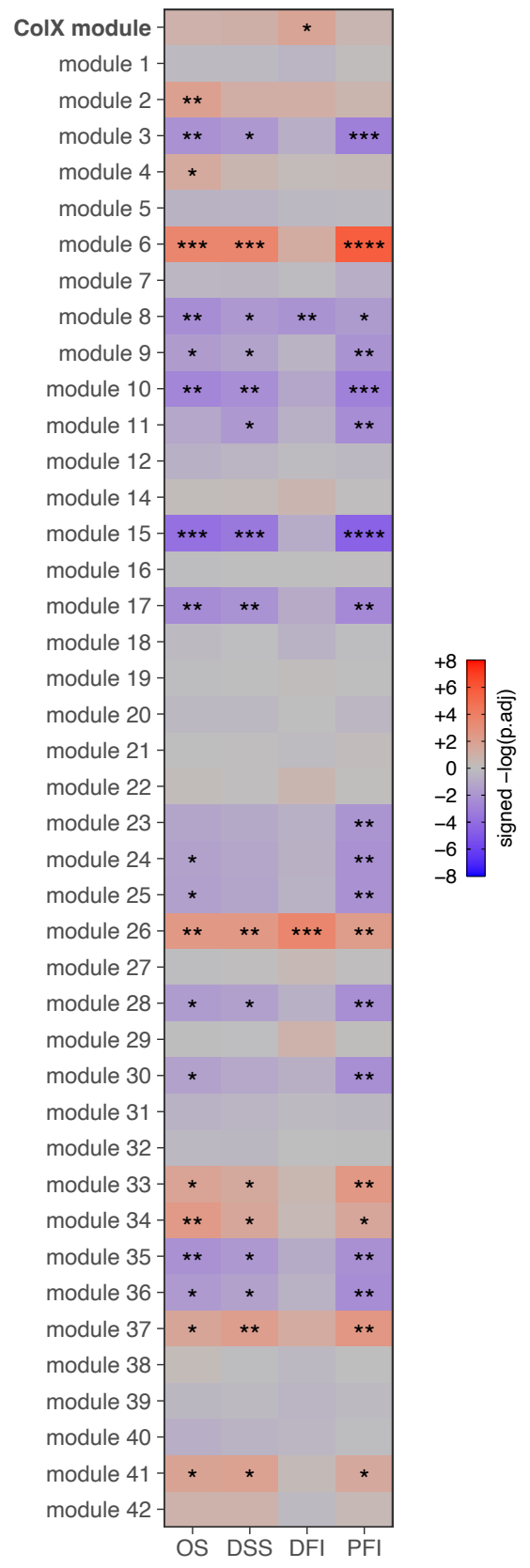**C****PAAD (M)**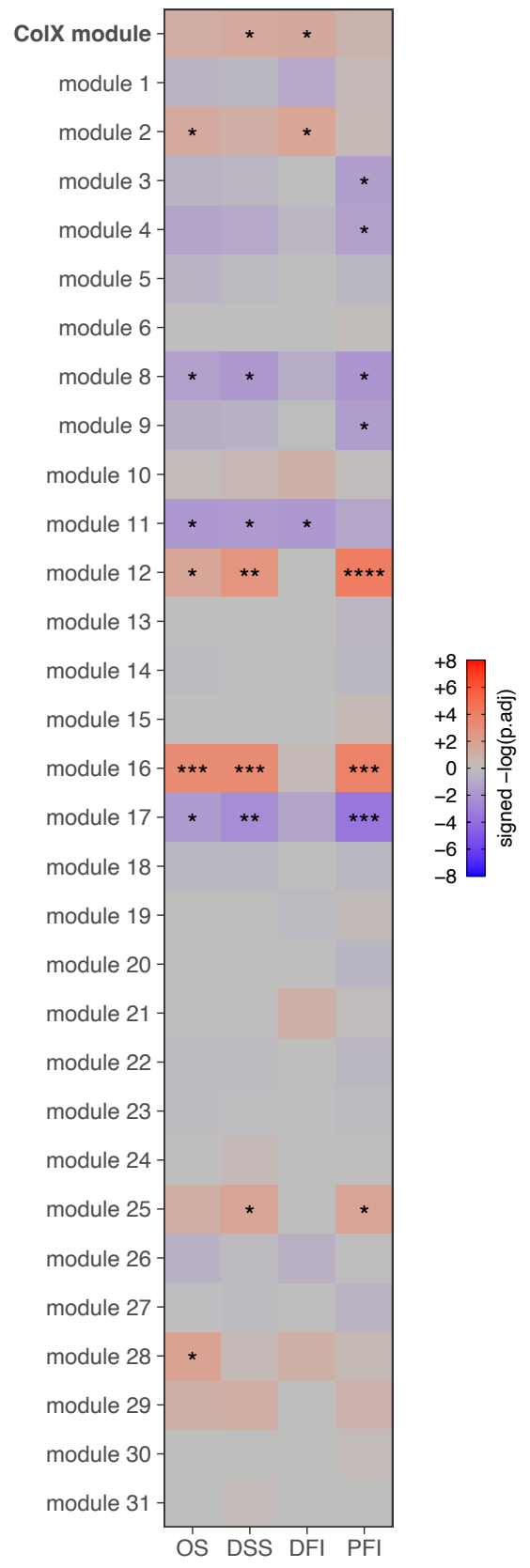**D****PAAD (F)**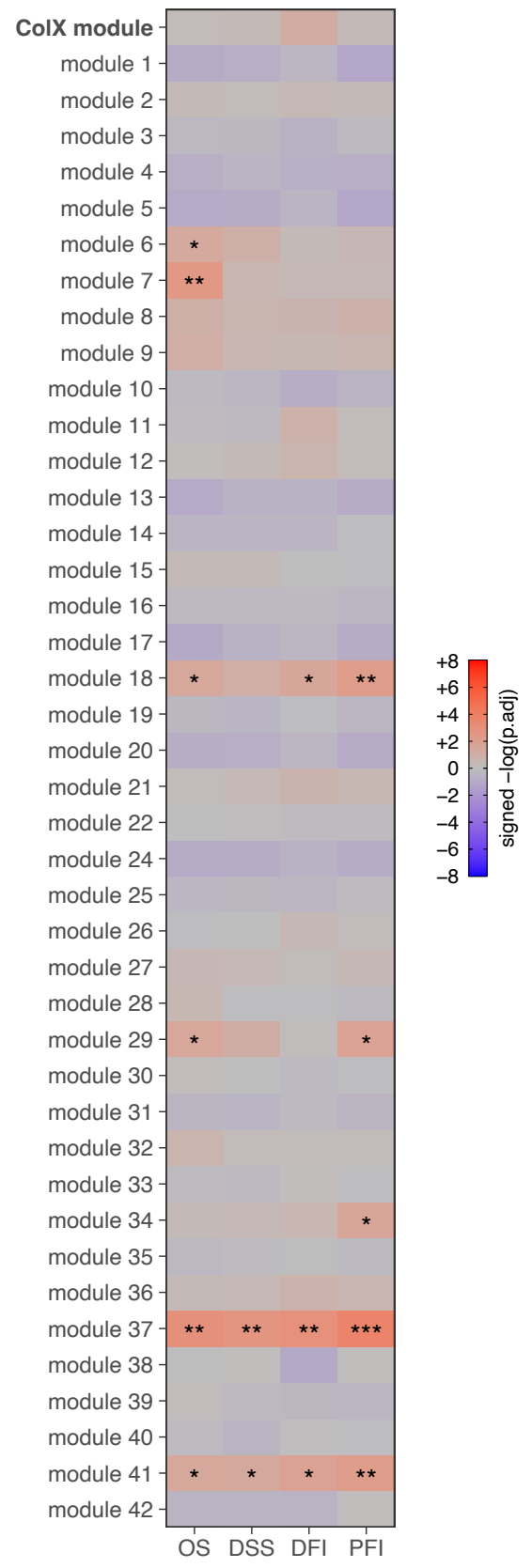

Supplement: Supplementary file 5 — Supplementary Material 5. Figure S5: TCGA WGCNA module expression correlates with variable survival risk. (A–D) BH-adjusted significance values for multivariate Cox proportional hazards models conditioning overall survival (OS), disease-specific survival (DSS), disease-free interval (DFI), or progression-free interval (PFI) on age, gender, binarized tumor stage, and module eigengene (ME) expression for WGCNA modules from (A) breast cancer, (B) pancreatic cancer, (C) male pancreatic cancer, and (D) female pancreatic cancer cohorts. ColX modules for each dataset are indicated by bolded labels (see Figure 4B for focused ColX module results). Significance values: *, p.adj < 0.05; **, p.adj < 0.01; ***, p.adj < 0.001; ****, p.adj < 0.0001. [file 12885_2025_13641_MOESM5_ESM.pdf]

**A****BRCA**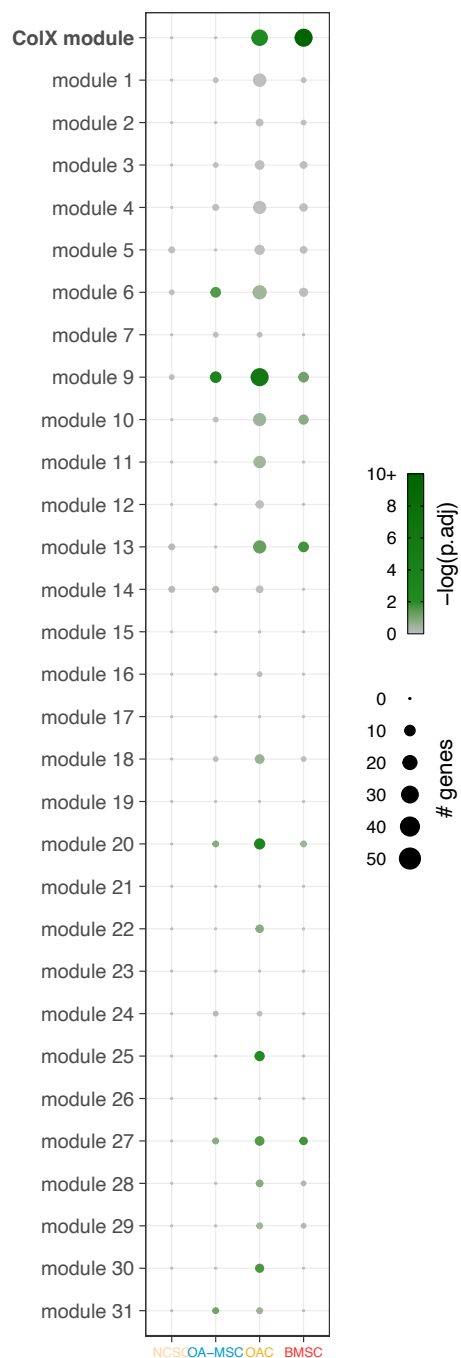**B****PAAD**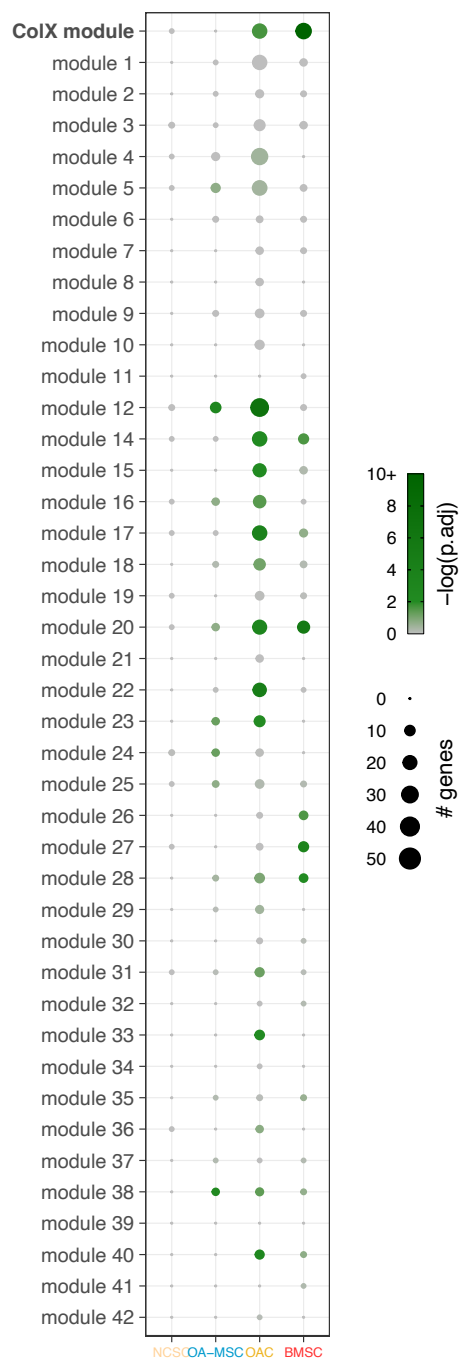**C****PAAD (M)**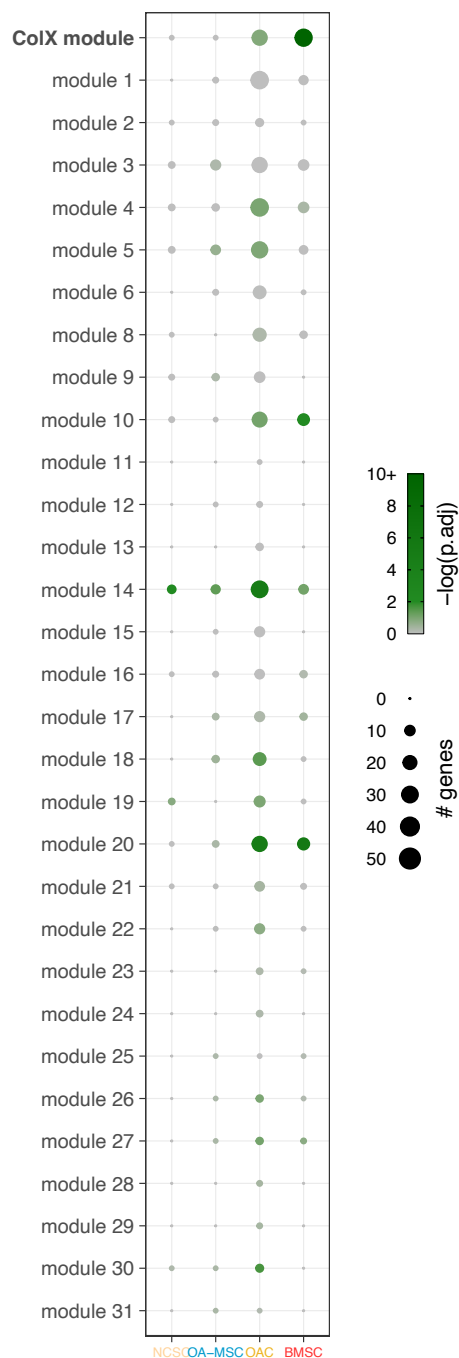**D****PAAD (F)**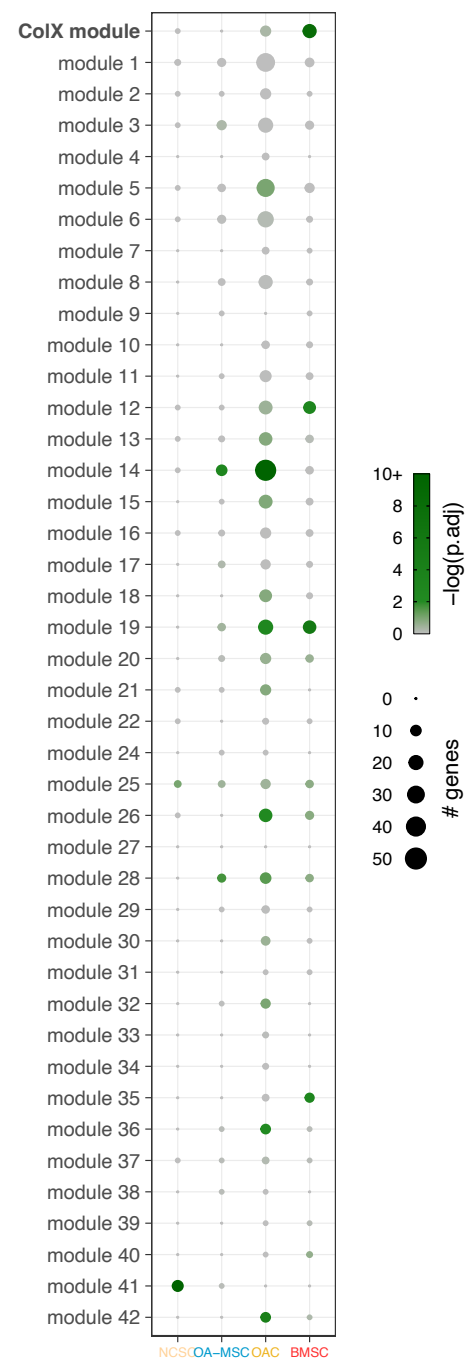**E****Gene set overlaps**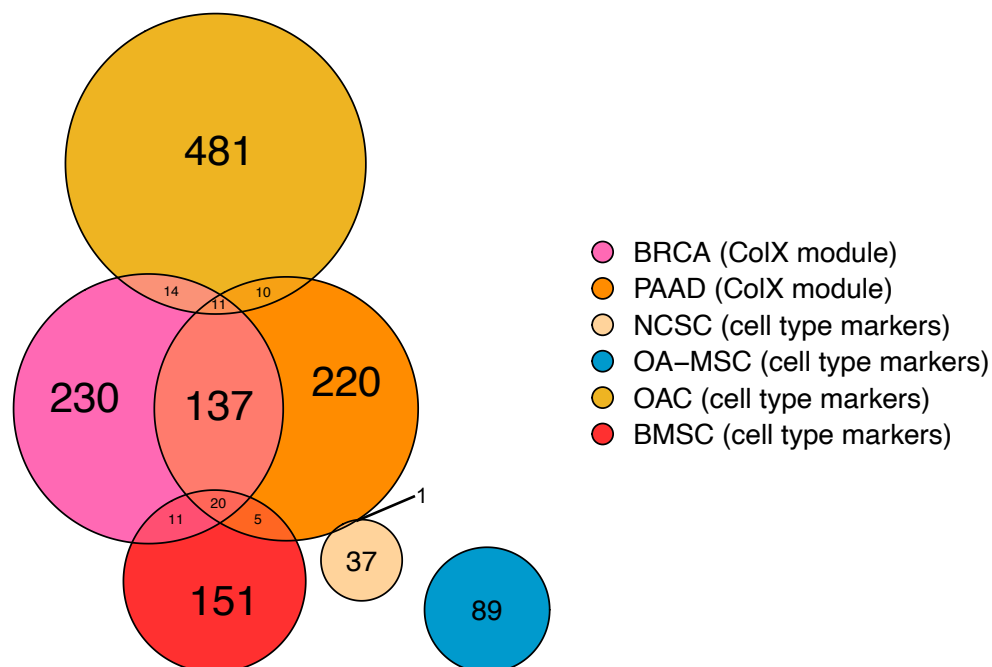**F****Gene set overlaps  
(EMT markers only)**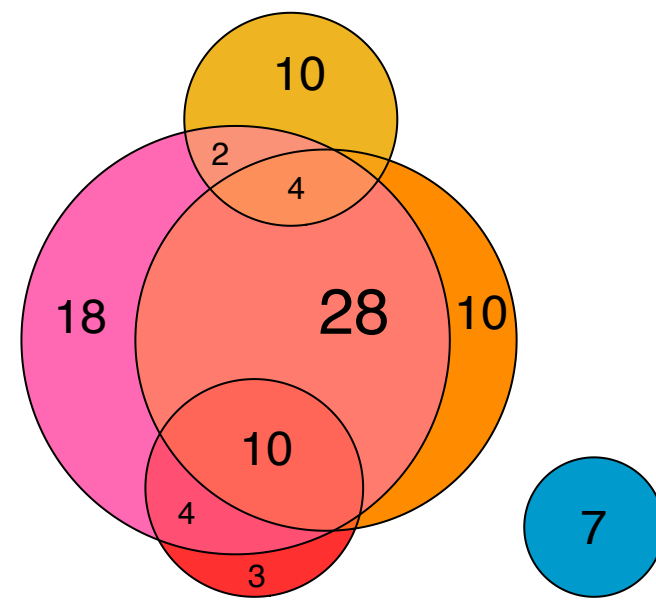

Supplement: Supplementary file 6 — Supplementary Material 6. Figure S6: Osteoarthritis cell type-specific markers are enriched for ColX module genes. (A–D) Bubble plots of OA cell type-specific marker gene enrichment in WGCNA modules from (A) breast cancer, (B) pancreatic cancer, (C) male pancreatic cancer, and (D) female pancreatic cancer cohorts. ColX modules for each dataset are indicated by bolded labels. (E) Overlap of genes within breast and pancreatic cancer ColX modules and OA cell type-specific gene sets. (F) Overlap of EMT pathway genes within breast and pancreatic cancer ColX modules and OA cell type-specific gene sets. Unlabeled sectors represent 0 gene overlap. NCSC is not shown as no NCSC-specific markers overlap with EMT pathway genes. NCSC, normal cartilage stromal cells; OA-MSC, osteoarthritis mesenchymal stromal cells; OAC, osteoarthritis chondrocytes; BMSC, bone marrow stromal cells. [file 12885_2025_13641_MOESM6_ESM.pdf]

**A**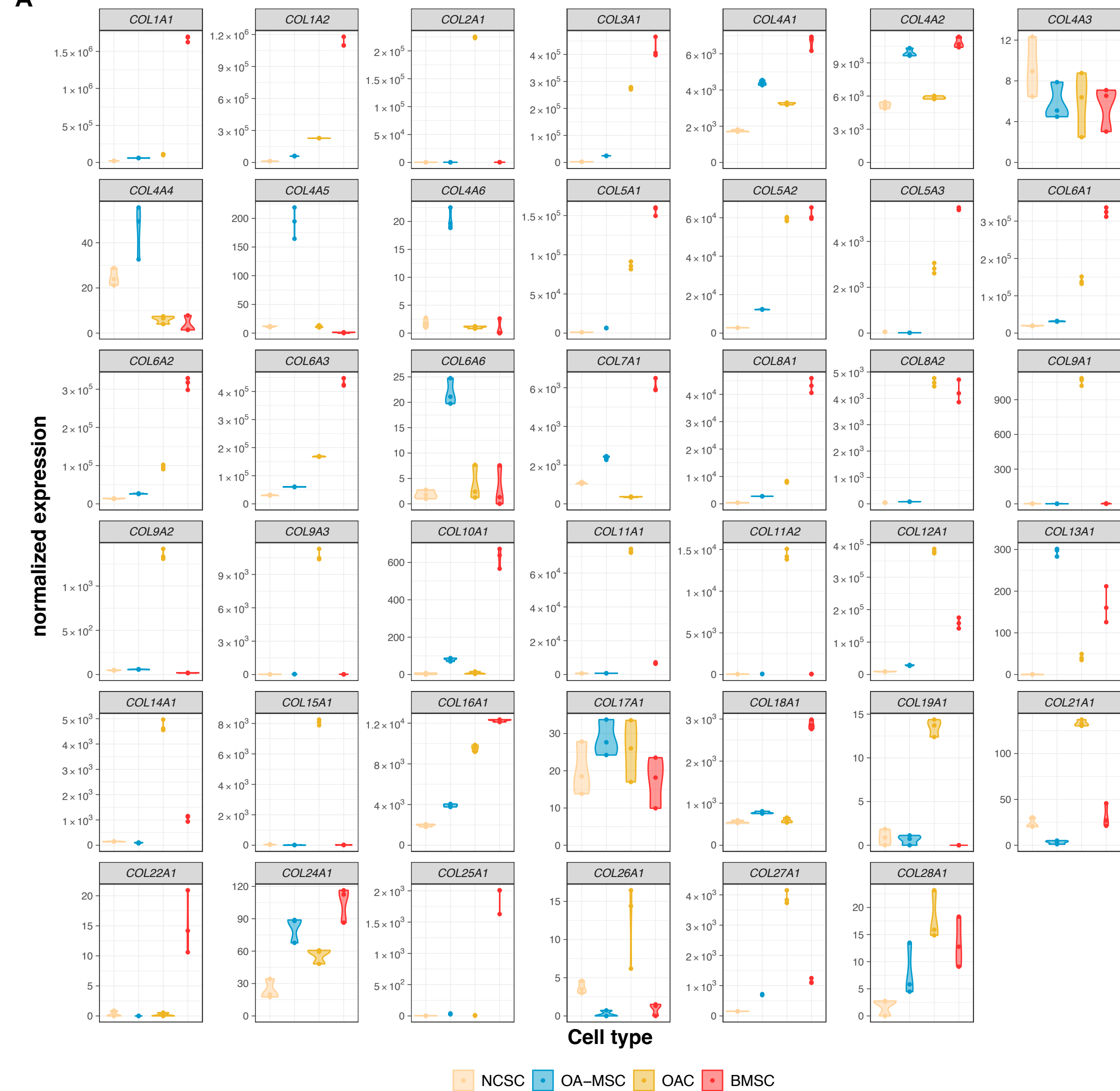**B**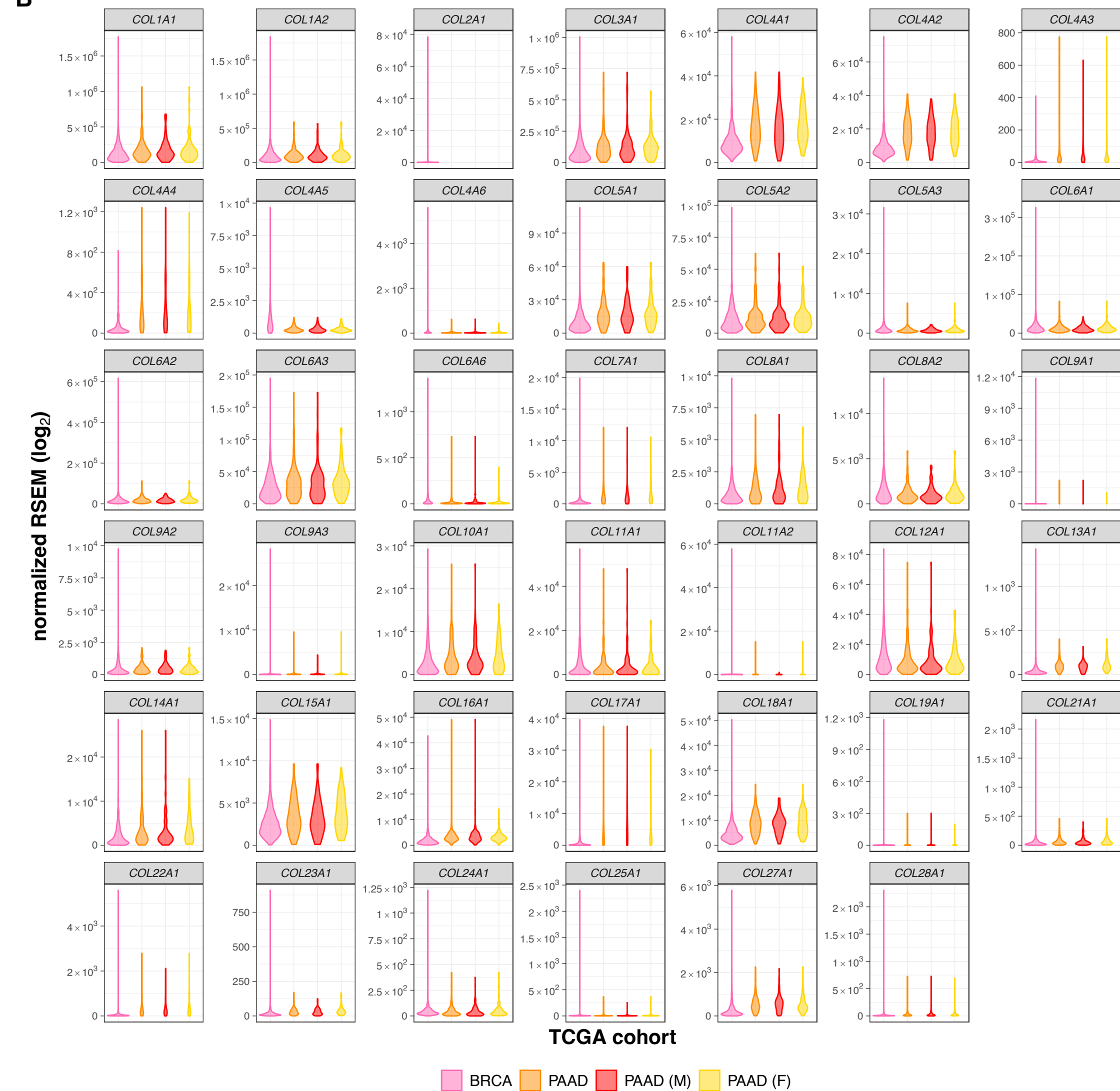

Supplement: Supplementary file 7 — Supplementary Material 7. Figure S7: Collagen gene expression varies across bone and cartilage cell types and TCGA cohorts. (A) Normalized expression of all collagen genes expressed at nontrivial levels in bone marrow and cartilage cell types. Note that COL6A4P1, COL6A5, COL20A1, and COL23A1 were filtered out as “low-expression” genes and are omitted here. NCSC, normal cartilage stromal cells; OA-MSC, osteoarthritis mesenchymal stromal cells; OAC, osteoarthritis chondrocytes; BMSC, bone marrow stromal cells. (B) Normalized expression of all collagen genes expressed at nontrivial levels in TCGA cohorts. Note that COL6A4P1, COL6A5, COL20A1, and COL26A1 were filtered out as “low-expression” genes and are omitted here; additionally, COL2A1 was only nontrivially expressed in BRCA. [file 12885_2025_13641_MOESM7_ESM.pdf]

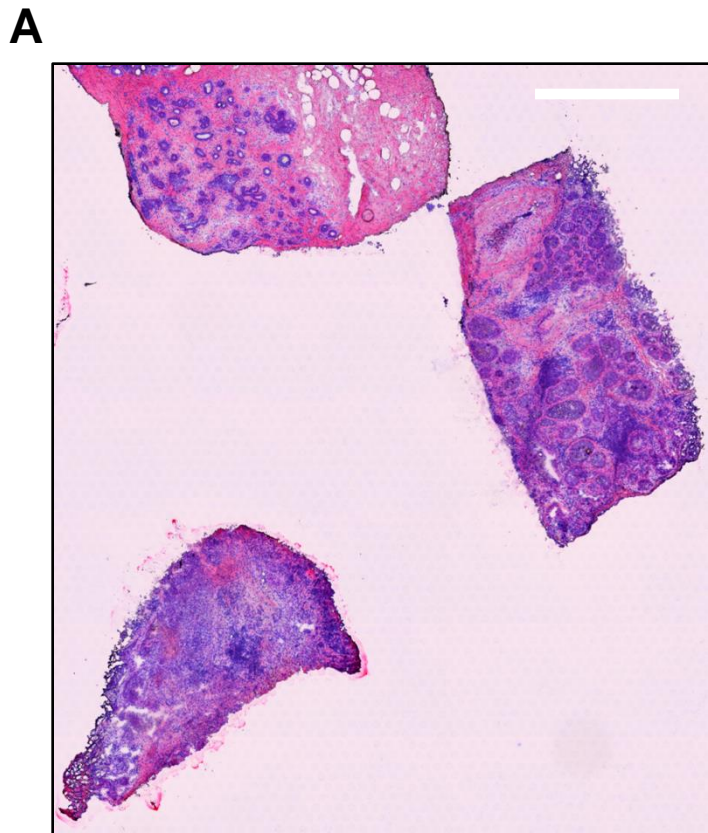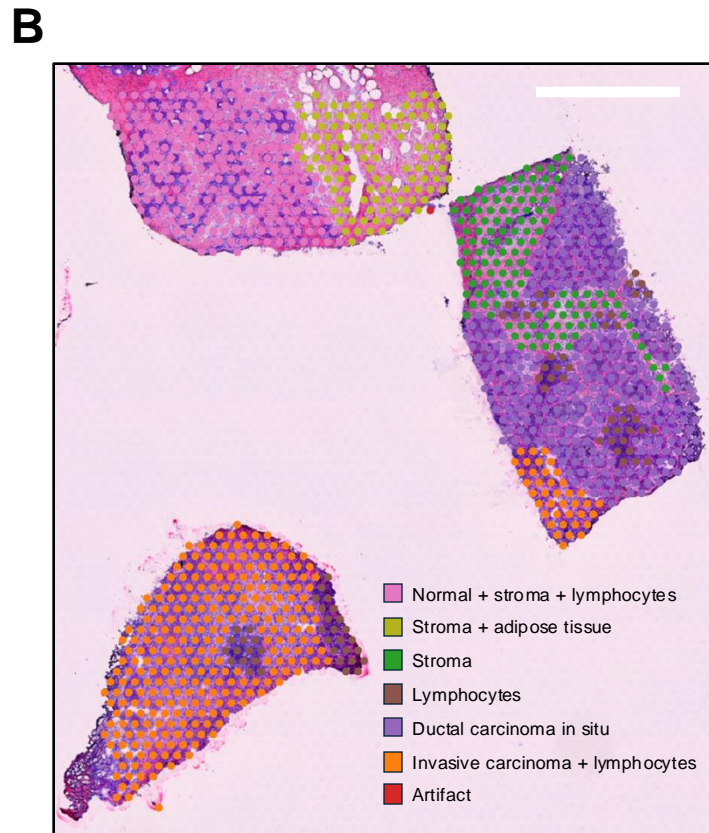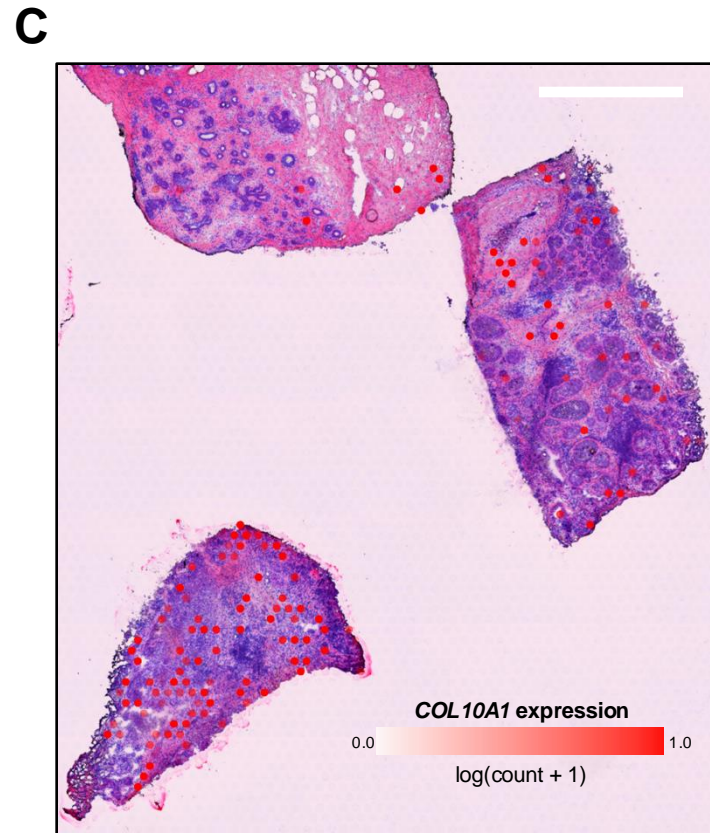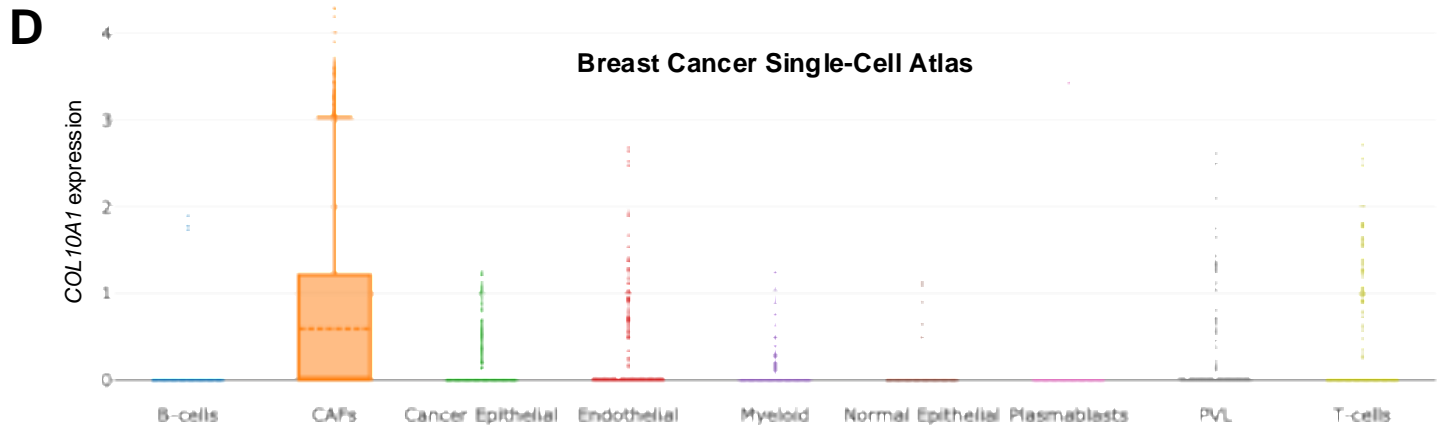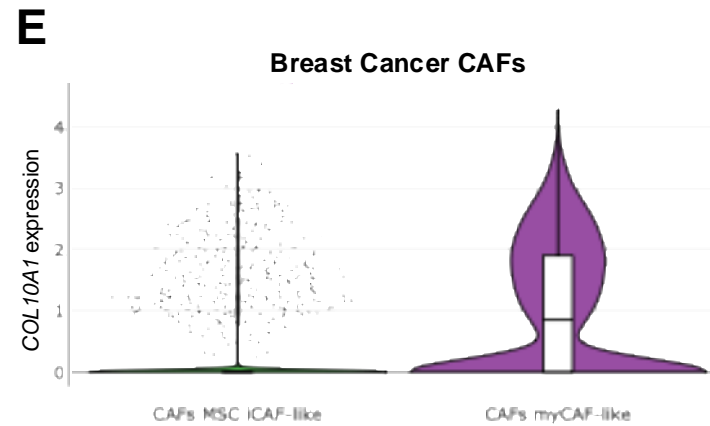

Supplement: Supplementary file 8 — Supplementary Material 8. Figure S8: A public single-cell and spatially resolved atlas of human breast cancers corroborates COL10A1 stromal predominance. (A–C) Spatial profiling of human breast cancer and normal tissue published by Wu et al. [97], demonstrating (A) H&E staining, (B) pathological classification, and (C) COL10A1 expression. Scale bars = 1 µm. (D and E) Single-cell COL10A1 expression across (D) all major cell types and (E) cancer-associated fibroblast (CAF) subsets in the breast cancer atlas. Data were obtained from (A–C) the UCSC Xena browser (accession Wu_Swarbrick_breast_cancer_CID44971) and (D and E) the Broad Institute Single-Cell Portal (accession SCP1039). PVL, perivascular-like cell; MSC iCAF, mesenchymal stem cell, inflammatory-like fibroblast. [file 12885_2025_13641_MOESM8_ESM.pdf]
