# Supplementary material for: Aberrant expression of collagen type X in solid tumor stroma is associated with EMT, immunosuppressive and pro-metastatic pathways, bone marrow stromal cell signatures, and poor survival prognosis
Source: BMC Cancer. 2025 Feb 12;25:247. doi: 10.1186/s12885-025-13641-y (PMC11823173; doi:10.1186/s12885-025-13641-y)
Supplement: Supplementary file 4 — Supplementary Material 4. Figure S4: TCGA WGCNA module expression tracks differential activity of immune, transcription factor, and cancer-associated fibroblast signatures. (A–P) Mean pathway activation (by QuSAGE) of various gene sets in samples with high module expression relative to samples with low module expression for each WGCNA module and TCGA cohort. (A–D) Results for cancer immunome genesets in (A) breast cancer, (B) pancreatic cancer, (C) male pancreatic cancer, and (D) female pancreatic cancer cohorts. Gene sets within each block are ordered by significance in respective ColX module; see Figure 3B for focused comparison. (E–H) Results for transcription factor target lists in (E) breast cancer, (F) pancreatic cancer, (G) male pancreatic cancer, and (H) female pancreatic cancer cohorts. Gene sets within each block are ordered by effect size in respective ColX module; see Figure 3C for focused comparison. (I–P) Results for cancer-associated fibroblast (CAF) gene sets derived from (I–L) breast tumor scRNA-Seq data and (M–P) pancreas/other tumor scRNA-Seq data, in (I and M) breast cancer, (J and N) pancreatic cancer, (K and O) male pancreatic cancer, and (L and P) female pancreatic cancer cohorts. Gene sets within each block are ordered by significance in respective ColX module; see Figures 3D and 3E for focused comparison. See Methods section for sample grouping process (note the same procedure for calculating G.A.M.E. and assigning “low” and “high” labels was applied to each WGCNA module respectively). Significance values were corrected across each module individually (by rows). Significance values: *, p.adj < 0.05; **, p.adj < 0.01; ***, p.adj < 0.001; ****, p.adj < 0.0001. [file 12885_2025_13641_MOESM4_ESM.pdf]

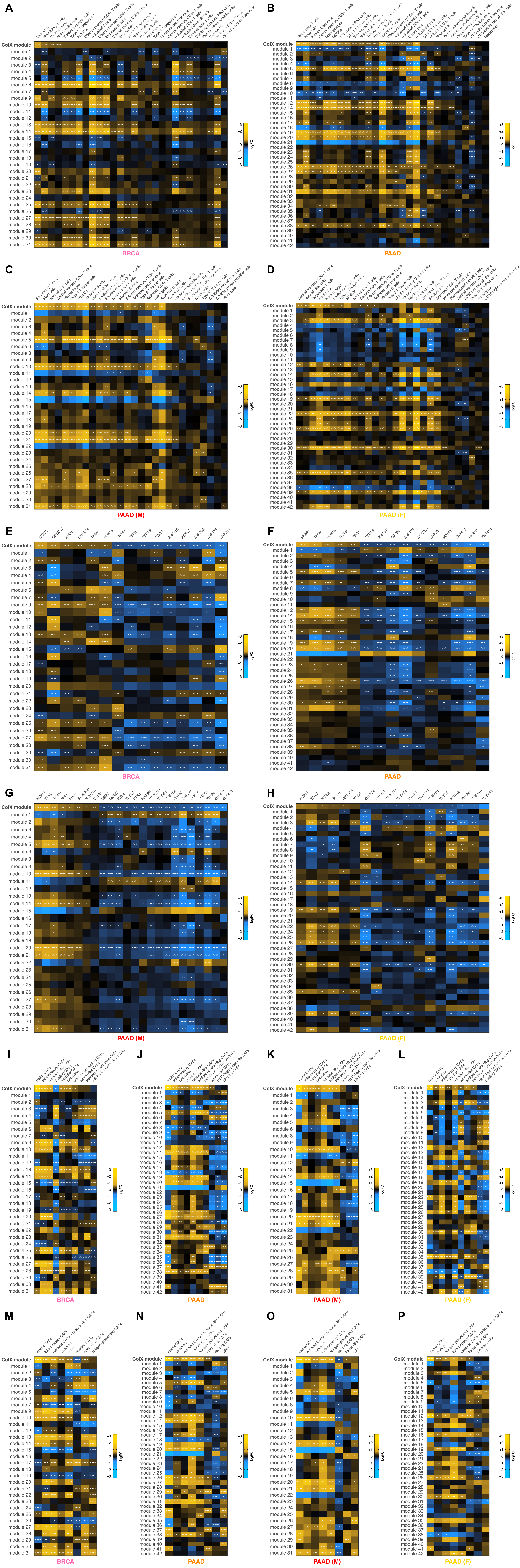
A
B
C
D
E
F
G
H
I
J
K
L
M
N
O
P
ColX module
logFC
BRCA
PAAD
PAAD (M)
PAAD (F)
